# Perinatal probiotic supplementation in the prevention of allergy related disease: 6 year follow up of a randomised controlled trial

**DOI:** 10.1186/s12895-015-0030-1

**Published:** 2015-08-01

**Authors:** Melanie Rae Simpson, Christian Kvikne Dotterud, Ola Storrø, Roar Johnsen, Torbjørn Øien

**Affiliations:** Department of Public Health and General Practice, Faculty of Medicine, Norwegian University of Science and Technology (NTNU), Postboks 8905, MTFS, 7491, Trondheim, Norway; Department of Dermatology, St Olavs Hospital, Trondheim University Hospital, Trondheim, Norway

**Keywords:** Allergy, Asthma, Atopic dermatitis, Paediatrics, Prevention, Probiotics, Rhinitis

## Abstract

**Background:**

Perinatal probiotics supplementation has been shown to be effective in the primary prevention of atopic dermatitis (AD) in early childhood, although the long term effects of probiotics on AD and other allergic diseases is less certain. We have previously reported a significant reduction in the cumulative incidence of AD at 2 years after maternal probiotic supplementation. In this study we present the effects of perinatal probiotics given to women from a general population on allergy related diseases in their offspring at 6 years.

**Methods:**

Four hundred and fifteen pregnant women were randomised to receive probiotic or placebo milk in a double-blinded trial from 36 week gestation until 3 months postpartum. Probiotic milk contained *Lactobacillus rhamnosos GG, L. acidophilus La-5* and *Bifidobacterium animalis* subsp. *lactis* Bb-12. At 6 years, children were re-assessed for AD, atopic sensitisation, asthma and allergic rhinoconjunctivitis (ARC).

**Results:**

At 6 years, 81 and 82 children were assessed for AD in the probiotic and placebo groups, respectively. In a multiple imputation analysis, there was as trend towards a lower cumulative incidence of AD in the probiotic group compared to the placebo group (OR 0.64, 95 % CI 0.39-1.07, *p* = 0.086; NNT = 10). This finding was statistically significantly in the complete case analysis (OR 0.48, 95 % CI 0.25-0.92, *p* = 0.027, NNT = 6). The prevalence of asthma and atopic sensitisation, and the cumulative incidence of ARC were not significantly affected by the probiotic regime at 6 years of age.

**Conclusions:**

Maternal probiotic ingestion alone may be sufficient for long term reduction in the cumulative incidence of AD, but not other allergy related diseases.

**Trial registration:**

ClinicalTrials.gov identifier: NCT00159523

**Electronic supplementary material:**

The online version of this article (doi:10.1186/s12895-015-0030-1) contains supplementary material, which is available to authorized users.

## Background

Atopic dermatitis (AD), asthma and allergic rhinoconjunctivitis (ARC) are a major cause of chronic disease in childhood. A revised version of the “hygiene hypothesis” suggests that the pattern of colonisation and the diversity of the intestinal microbiota may be an important factor in the increased prevalence of these diseases observed over the past several decades [1–3]. Subsequently, probiotics have been investigated in the prevention and treatment of allergy related diseases [3–8], with the strongest evidence emerging for the primary prevention of atopic dermatitis [3–5]. Throughout this paper we refer AD, asthma and ARC as “allergy related diseases”, recognising that not all presentations of these conditions are related to a classic IgE-mediated inflammatory process.

Randomised controlled trials (RCTs) testing probiotics in the prevention of childhood allergy related disease are heterogeneous and have used a variety of bacterial strains, administration regimes and varying ages of follow-up. Using information from the first published follow up for each trial, a recent meta-analysis concluded that probiotic administration is protective against the development of AD in infancy [3]. Among the studies with follow-up at or beyond 5 years of age, the greatest protective benefit of probiotics against AD appears to be in early childhood and it is less certain if this effect persists until school age [9–25]. Only one of these studies did not specify a maternal or family history of atopy as an inclusion criteria [9, 10], and there is therefore a particular need for further longer term follow-up studies to determine the ongoing effect of perinatal probiotics in general populations.

The Probiotics in the Prevention of Allergy among Children in Trondheim (ProPACT) study is a double-blinded RCT investigating the effect of maternal probiotic supplementation on childhood allergy related diseases in a general population. The initial results of the ProPACT study demonstrated a clinically significant reduction in the cumulative incidence of AD at 2 years (OR 0.51, 95 % CI 0.30 – 0.87, *p* = 0.013), with the greatest reduction seen in children not considered at “high risk” for allergy related disease based on a negative family history [26]. Probiotic supplementation did not significantly affect the incidence of asthma, ARC or atopic sensitisation at 2 year of age, although the diagnosis of the former two diseases is uncommon and controversial at such a young age.

The participating children were re-contacted and re-assessed at 6 years of age for the presence of allergy related diseases and allergic sensitisation. The aim of the current paper was to investigate the effect of maternal perinatal probiotic supplementation on the cumulative incidence of AD and ARC and prevalence of asthma and atopic sensitisation at 6 years of age.

## Methods

### Participants and design

The ProPACT study has been described in detail previously [26]. Briefly, 415 women living in Trondheim, Norway, were randomised to receive daily probiotic supplementation or placebo from 36 weeks gestation until 3 months postpartum. Probiotic supplementation consisted of 250 mL of low fat fermented milk containing 5 × 10^10^ colony-forming units (CFUs) of *Lactobacillus rhamnosus GG* (LGG) and *Bifidobacterium animalis* subsp. *lactis* Bb-12 (Bb-12) and 5 × 10^9^ CFU of *L. acidophilus* La-5 (La-5). The equivalently tasting placebo milk was sterile and contained no probiotic bacteria. The study milk was consumed by the women both pre- and postnatally, and the children did not receive any probiotic supplementation as a part of this study.

Participants were requested to complete questionnaires regarding lifestyle factors during pregnancy and at 6 weeks, 1 year and 2 years postpartum. These questionnaires detailed family history of allergy related diseases, dietary and parental smoking habits, housing conditions, family structure and the general health of the children. A child health questionnaire focusing on the presence of allergic symptoms and antibiotic use was completed at 1, 2 and 6 years. Information regarding mode of delivery was unavailable, although assumed to be unaffected by the intervention regime. Those who had not initially responded to the 6 year child health questionnaire were re-sent this questionnaire in October 2013, when the children were between 8 and 10 years of age and responders to this re-sent questionnaire were considered “late responders”.

Families were encouraged to attend a clinical examination for AD prior to 1 year if the child developed an itchy rash which lasted for more than 4 weeks. At 2 and 6 years, all children were invited to attend a clinical examination, including a structured interview, and allergy testing consisting of skin-prick testing (SPT) and specific IgE (sIgE). This examination was conducted by specially trained nurses at the 6 year follow-up who were unaware of treatment allocation. Participants were un-blinded after the publication of the 2 year follow-up results [26]. Prior to this, the participants and investigators were blinded to treatment allocation which was conducted by the Department of Applied Clinical Research at the Norwegian University of Science and Technology through a computer-generated randomisation list without restrictions.

The trial was approved by the Regional Committee for Medical Research Ethics for Central Norway (Ref. 097–03) and the Norwegian Data Inspectorate (Ref. 2003/953-3 KBE) and the trial protocol is registered in ClinicalTrials.gov (identifier NCT00159523). Parents gave their written consent.

### Outcomes

The primary outcomes of interest were: cumulative incidence of atopic dermatitis (AD) and allergic rhinoconjunctivitis (ARC), and the 12 month prevalence of asthma. Children who attended the clinical examination(s) were assessed for AD using the UK Working Party (UKWP) diagnostic criteria [27]. Children who were assessed under these criteria as having AD at any point up to 6 years were considered to ever have had AD in the cumulative incidence estimate.

The cumulative incidence of ARC was defined by a positive answer to the question “Has your child *ever* had hay fever or allergic rhinoconjunctivitis?” in the 1, 2 or 6 year questionnaire. Current asthma was defined as a positive answer to both questions “Has your child *ever* been diagnosed with asthma by a doctor?” and “In the past 12 months, has your child been treated with tablets, inhalers or other medications for wheezing, chest tightness or asthma?”. These questionnaire based definitions were used in order to minimise the proportion of participants with missing data and vary from the clinical examination based definitions presented in the 2 year follow-up article (Additional file 1: Table S1). Prompted by the findings of other probiotic trials, we include here the cumulative incidence of wheeze defined by a positive answer to both questions “Has your child *ever* had whistling in the chest?” and “Has your child *ever* had episodes of wheezing or tightness in the chest?” and a history of a lower respiratory tract infection, subcategorised into bronchitis and pneumonia, in the 1, 2 or 6 year questionnaire.

Atopic sensitisation was assessed as a secondary endpoint and was defined as any SPT wheal ≥ 3 mm or any sIgE level ≥ 0.35kUL^−1^. Both SPT and sIgE testing was performed as described previously [26] according to the ISAAC II procedure [28] for the following allergens: mite, mould, cat and dog dander, birch, timothy (grass) and mugwort pollen, egg white, codfish, hazelnut, peanut and cow’s milk.

### Statistical analysis

All statistical analyses were conducted using Stata/IC 13.1. The sample size calculation and randomisation procedure has been previously reported [26]. Due to the presence of missing data, the intention to treat (ITT) analysis strategy [29] included a main analysis using multiple imputations by chained equations (MICE) under the assumption that the data is missing at random (MAR) and a pattern mixture model (PMM) analysis to assess the sensitivity of the conclusions to this assumption. One hundred (m = 100) imputed data sets were created for the MICE analysis using the following predictor variables: treatment allocation, family history of atopy, siblings, sex, paternal smoking, antibiotic use within the first year of life, parentally reported eczema, protocol compliance and disease outcomes at 2 and 6 years of age. Further details regarding the MI model are provided in Additional file 1. A complete case analysis is also presented which includes participants who submitted the 6 year health questionnaire and or attended the 6 year clinical interview.

Univariate logistic regression was used to assess the impact of maternal probiotic supplementation on each of the outcomes for both multiple imputed datasets and complete case analyses. Although potential confounders should be balanced in an RCT, an alternate logistic regression model including family history, sex and siblings was assessed because of their previously reported associations with allergic disease and slight imbalance in the latter two.

A missing not at random (MNAR) sensitivity analysis was conducted using the pattern-mixture model version of the user written Stata command *rctmiss*. A detailed explanation of this analysis is provided in the Additional file 1.

## Results

### Participants

**Fig. 1:**
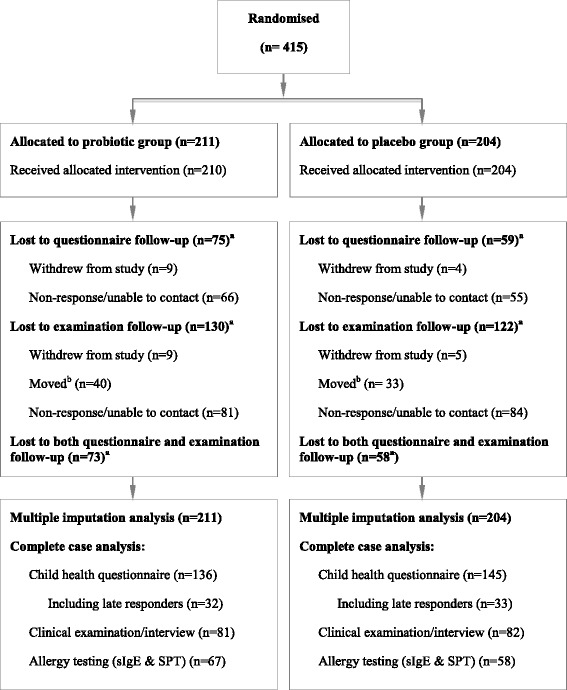
Participant flow diagram. The exact number of women who were invited and or assessed for eligibility is not available. ^a^Several children were lost to both questionnaire and examination follow-up as displayed at the bottom of this box. ^b^Number of participants who moved from the Trondheim municipality was estimated from public address catalogues

Participating families were recruited between September 2003 and September 2005, and the initial 6 year follow up occurred from December 2009 to December 2011. The 6 year child health questionnaire was completed by 281 (67.7 %) families and 163 (39.3 %) attended the clinical interview (Fig. 1), with no significant difference in attendance rates between the probiotic and placebo groups. Mean age of follow-up at the clinical examination was 6.3 years (SD 0.2 years) in both probiotic and placebo arms. The mean age of completion of the questionnaire was also comparable between treatment arms among both initial and late responders (data not shown).

The baseline data and characteristics of participants randomised to receive probiotic supplementation or placebo showed minimal differences between the treatment groups (Table 1). Differences considered to be of potential influence due to reported association with childhood allergic diseases included the higher proportion of males (49.7 vs 41.8 %) and children with older siblings (44.0 vs 39.0 %) in the probiotic group compared to the placebo group.

**Table 1:** Baseline data, characteristics and allergy related disease at 2 years

	Comparison of treatment groups				Comparison of drop out cases				
	Probiotic (*n* = 211)		Placebo (*n* = 204)		Attended 6 yr examination		Drop-outs		
	n^a^		n^a^		n^a^		n^a^		*p*-value^b^
Baseline data and characteristics									
Age, mother (years), mean ± SD	191	30.1 (3.9)	189	30.3 (4.4)	162	30.7 (3.9)	218	29.8 (4.2)	0.024
Education, mother (yrs), mean ± SD	183	15.3 (2.2)	181	15.2 (2.3)	156	15.2 (2.1)	208	15.2 (2.4)	0.968
Education, father (yrs), mean ± SD	184	14.8 (2.7)	179	14.7 (2.4)	156	14.7 (2.5)	207	14.8 (2.6)	0.561
Birth weight (g), mean ± SD	187	3662 (478)	176	3596 (474)	161	3626 (466)	202	3635 (486)	0.814
Sex (male), n (%)	193	96 (49.7)	191	80 (41.8)	163	76 (46.6)	221	100 (45.3)	0.151
Premature, n (%)	189	5 (2.7)	185	7 (3.8)	162	5 (3.1)	212	7 (3.3)	1.000^c^
Siblings, n (%)	207	91 (44.0)	200	78 (39.0)	163	82 (50.3)	244	87 (35.7)	0.003
Atopy in family, n (%)	207	152 (73.4)	200	148 (74.0)	163	127 (77.9)	244	173 (70.9)	0.115
Smoking mother^d^, n (%)	204	16 (7.8)	200	19 (9.5)	163	13 (8.0)	241	12 (9.1)	0.686
Smoking father^d^, n (%)	203	35 (17.2)	198	38 (19.2)	163	21 (12.8)	238	52 (21.8)	0.022
Breastfed ≥ 3 months, n (%)	168	164 (97.6)	166	162 (97.6)	155	153 (98.7)	179	173 (96.7)	0.293^c^
At least one pet at home^d^, n (%)	207	52 (25.1)	200	53 (26.5)	163	47 (28.8)	244	58 (23.7)	0.253
Used antibiotics^d^, n (%)	161	35 (21.7)	159	34 (21.4)	152	34 (22.4)	168	35 (20.8)	0.739
Fish ≤ 6 mo., n (%)	163	35 (21.5)	165	27 (16.4)	155	22 (14.2)	173	40 (23.1)	0.039
Vegetables ≤ 6 mo., n (%)	166	84 (50.6)	166	105 (63.3)	156	90 (57.7)	176	99 (56.3)	0.791
Protocol compliance^e^, n (%)	150	130 (86.7)	148	128 (86.5)	147	134 (91.2)	151	124 (82.1)	0.022
Allergy related disease at 2 years									
Atopic dermatitis, n(%)	138	29 (21.0)	140	48 (34.3)	145	52 (35.9)	133	25 (18.8)	0.001
Allergic sensitisation^f^, n(%)	131	20 (15.3)	133	15 (11.3)	140	26 (18.6)	124	9 (7.3)	0.007

^a^N: number of observed cases for each variable differs based on the source of information; ^b^p-values for attendees versus dropouts calculated using t-test for continuous variables and χ^2^ for binary variables; ^c^Fisher’s exact test used to calculate *p*-values for binary variables with frequency <=5 in one or more cell of the contingency table; ^d^Exposure to smoking, household pets or antibiotics reported anytime during the first year of life or during pregnancy; ^e^Compliance defined as consumption of 250 mL of study on ≥50 % of days, no consumption of other probiotic products and breast feeding for ≥ 3 months; ^f^Positive skin prick test and or specific IgE level, if only one negative test result was available, the child was considered not sensitised

### Characteristics of participants followed-up at 6 years

Compared to those lost to clinical follow up, the children who attended the 6 year clinical examination had older mothers, were less likely to have a father who smoked during their first year of life and to have received fish before 6 months of age, and were more likely to have a family history of atopy, an older sibling and study protocol compliance (Table 1). Additionally, the children sensitised and or diagnosed with AD at 2 years were more likely to have attended the 6 year examination (Table 1). Similar differences were observed between children with and without a completed 6 year questionnaire (details provided in Additional file 1: Table S2).

### Atopic dermatitis

A trend towards a lower cumulative incidence of UKWP diagnosed AD in the probiotic group with an odds ratio (OR) of 0.64 (95 % CI 0.39-1.07, *p* = 0.086; NNT = 10) was observed in the MICE analysis (Table 2). This finding was statistically significantly in the complete case analysis (OR 0.48, 95 % CI 0.25-0.92, *p* = 0.027; NNT = 6). The adjustment for the covariates family history, sex and presence of older siblings resulted in inconsequential changes in the calculated OR estimates and did not substantially improve the precision of the estimate (data not shown).

**Table 2:** Prevalence and cumulative incidence at 6 years of allergy related diseases, wheeze and lower respiratory tract infections in the probiotic and placebo groups

	Imputed estimates			Observed cases				
Allergy related disease	Probiotic, n=211	Placebo, n=204	Odds ratio (95 % CI)^a^	Probiotic		Placebo		Odds ratio
	% (95 % CI)	% (95 % CI)		n/n	% (95 % CI)	n/n	% (95 % CI)	(95 % CI)^a^
Current disease								
Asthma	2.3 (0.0-4.7)	0.9 (0.0-2.5)	1.68 (0.21-13.20)	3/136	2.2 (0.7-6-7)	1/145	0.7 (0.0-4.8)	3.25 (0.33-31.6)
Allergic sensitisation	30.0 (21.2-38.8)	28.0 (18.8-37.1)	1.11 (0.62-1.96)	23/80	28.8 (19.7-39.8)	19/78	24.4 (16.0-35.3)	1.25 (0.62-2.54)
Cumulative incidence								
Atopic dermatitis	29.3 (21.2-37.4)	39.1 (30.2-48.0)	0.64 (0.39-1.07)^b^	22/81	27.2 (17.3-37.1)	36/82	43.9 (32.9-54.9)	0.48 (0.25-0.92)^c^
ARC	21.6 (14.6-28.6)	18.8 (12.0-25.7)	1.19 (0.66-2.16)	22/134	16.4 (10.1-22.8)	20/145	13.8 (8.1-19.5)	1.22 (0.64-2.37)
Wheeze	39.0 (30.9-47.1)	45.8 (37.4-54.1)	0.75 (0.47-1.22)	46/132	34.9 (26.7-43.0)	55/142	38.7 (30.7-46.8)	0.85 (0.52-1.38)
LRTI (any)	30.6 (22.2-39.1)	36.8 (29.1-44.5)	0.76 (0.47-1.23)	33/128	25.8 (18.1-33.4)	40/138	29.0 (21.4-36.6)	0.85 (0.50-1.46)
Bronchitis	23.6 (16.3-30.9)	27.6 (20.2-35.0)	0.81 (0.48-1.36)	29/130	22.3 (15.1-29.5)	32/139	23.0 (16.0-30.1)	0.96 (0.54-1.70)
Pneumonia	10.2 (4.5-15.9)	15.4 (9.4-21.4)	0.61 (0.29-1.32)	7/129	5.4 (1.5-9.4)	17/141	12.1 (6.6-17.5)	0.42 (0.17-1.04)^d^

ARC: allergic rhinoconjunctivitis; LRTI: Lower respiratory tract infection; ^a^Unadjusted logistic regression odds ratio; Significant or near significant *p*-values: ^b^ *p*=0.086, ^c^ *p*=0.027 and ^d^ *p*=0.062

### Allergic rhinoconjunctivitis, asthma, sensitisation and lower respiratory tract infections

There was no statistically significant difference observed between the cumulative incidence of ARC, 12 month prevalence of asthma or current atopic sensitisation in the MICE or complete case analyses (Table 2). Parental reported cumulative incidence of wheeze and lower respiratory tract infections were not influenced by the probiotic regime in observed cases or imputed estimates. There was a trend towards a lower cumulative incidence of pneumonia in the probiotic group (OR 0.41, 95 % CI 0.17-1.04, *p* = 0.062, Table 2) which was not statistically significant in the MICE analysis.

### Sensitivity analysis

The results of the sensitivity analysis are provided in the Additional file 1. Briefly, if children with AD were more likely to be lost to follow-up in the probiotic group than the placebo group, then the preventative effect of probiotics would have been weakened.

## Discussion

Maternal probiotic supplementation given to a general population of women appears to have an ongoing preventative effect on the cumulative incidence of AD until school age, however this did not reach statistical significance in the MICE analysis. There was no observed effect of probiotics on the cumulative incidence of ARC, the 12 month prevalence of asthma or current atopic sensitisation.

A significant proportion of cases of allergy related diseases occur in children who are otherwise considered not to be at “high risk” and therefore primary prevention strategies must also be assessed in general populations [30]. This long term follow-up of the ProPACT trial is an important addition to the literature concerning probiotics in the prevention of allergic disease as one of few studies to recruit participants from a general population [7, 8, 29]. The only other RCT to have reported long term follow-up in a general population observed no benefit of their probiotic regime on the prevalence of any allergic disease or the cumulative incidence of questionnaire defined AD at 8–9 years of age [9, 10]. Their regime included *Lactobacillus paracasei* spp *paracasei* F19 supplementation given to children during weaning. In comparison, our study involved pre- and postnatal supplementation of a mixture of probiotics which included the *L. rhamnosus GG* (LGG) strain. Both LGG and pre- and postnatal regimes were found to be associated with reduced RR of AD on sub-group analysis in a meta-analysis [3]. Additionally, we report UK Working Party diagnostic criteria defined AD which is a more extensively validated method of diagnosis [31, 32].

Looking to other trials, five RCTs have assessed the cumulative incidence of AD at 5 years of age or beyond in “high risk” populations [11–23]. The trial presented by Kalliomaki et al. [11–13] and the *Lactobacillus rhamnosus* (HN001) arm of the study by Wickens et al. [14–16] report a significant reduction in the cumulative incidence of AD, which is sustained, although reduced, at follow-up at 6 years. Together with the current study, these trials indicate that the beneficial effect of probiotics on AD is most pronounced in infancy and may continue into early childhood. Furthermore, they suggest that we are observing a true primary preventative effect, rather than an intervention which delays the onset of AD. Contrastingly, the preventative effect of probiotics seen at 2 years in the large RCT published by Kukkonen/Kuitunen et al. [17, 18] showed no trend towards ongoing benefit on follow-up at 5 years. The *Bifidobacterium animalis* subsp. *lactis* (HN019) arm of Wickens et al. [14–16] and 2 other trials [19–23] demonstrated no significant effect of probiotics on the cumulative incidence of AD at any follow-up time point from 1 to 7 years of age. It is interesting to note that all of the studies with an observed ongoing effect administered a *L. rhamnosus* strain pre- and postnatally, and that if the child was breastfed, the postnatal supplementation was given solely to the mother during the first months with the exception of Kalliomaki et al. [11–13] where approximate 57 % of participants opted to give the probiotic or placebo capsules directly to the newborn children. In contrast, studies without an ongoing effect have either administered probiotic species other than *L. rhamnosus* and or specified that the probiotic supplements were to be given directly to the children regardless of breastfeeding*.* Further research is required to investigate if these observations are coincidental or represent strain and or regime specific effects.

The lack of effect on asthma, ARC and atopic sensitisation may reflect a true lack of effect or that the current study is under-powered to observed smaller differences in less frequent diseases. This is a universal problem for probiotic trials reporting asthma as an outcome [8]. Two recent meta-analyses concluded that there is not enough evidence to support perinatal probiotic supplementation in the prevention of childhood asthma or wheeze [7, 8]. Reassuringly, neither our study nor these meta-analyses found that probiotics increased the risk of asthma or wheeze, a concern which arose after long term follow-up by Kalliomaki et al. [8, 11] Whilst one of these meta-analyses suggested that probiotics may increase lower respiratory tract infections [8], our study does not support this conclusion. On the contrary, we observe a trend towards lower cumulative incidence of pneumonia in the probiotic group. Atopic sensitisation was found to be significantly reduced in a sub-group meta-analysis of regimes which combined pre- and postnatal administration [7]. Consistent with the ProPACT study, none of the individual longer term follow-up studies have observed a significant effect of probiotics on sensitisation at school age. Interestingly, *Lactobacillus acidophilus* was found to be associated with an increased rate of atopic sensitisation in a multivariate meta-regression analysis [7]. This observation requires further investigation and may, in part, explain the lack of effect of the probiotic regime on sensitisation in the ProPACT trial.

The major limiting factor of this RCT is the high proportion of missing data which naturally raises concerns regarding the generalisability of the results and introduction of bias. Following the four point ITT analysis strategy recommended by White et al. [29] we have attempted to follow-up all participants, performed a primary analysis using MICE and a sensitivity analysis using PMM. Both of the latter two models account for all randomised participants under a range of assumptions about the cause of missingness and in doing so attempt to minimise bias from covariate-related and outcome-related drop-out, respectively. The reasons for loss to follow-up are primarily unknown, however very few participants actively withdrew from the study. An estimated 73 participants had moved from the study region, which would have precluded their attendance at the clinical examination in a presumably random manner. A number of these participants were followed-up through the questionnaire. In terms of generalisability, the original ProPACT population was similar to the total PACT population, which in turn was representative of the general population in Trondheim, Norway [26]. At the 6 year follow-up, the remaining participants were more likely to have a family history of atopy, older siblings and a pet and less likely to have a father who smoked. As these differences were not large we believe that the results are still generalisable to westernised populations where there is a reasonably high rate of allergy related disease. The PMM sensitivity analysis is particularly pertinent in this case because atopic sensitisation and or a diagnosis of AD at 2 years are associated with both attendance at the 6 year clinical examination and a diagnosis of AD at 6 years. This raises suspicions that the data is partially MNAR which would lead to biased estimates under both the complete case and multiple imputations analysis models. The PMM analysis suggests that results of this study for ARC, asthma and atopic sensitisation would have only been affected if there was an unrealistically strong association between disease and missingness in a single treatment arm. On the other hand, the magnitude of the preventative effect attributed to probiotics for AD at 6 years must be considered with caution, as the observed benefit is sensitive to the assumption that the relationship between AD and loss to follow-up is essentially identical in both the probiotic and placebo groups. Regardless of whether the outcome variables are partially MNAR, the MICE estimates are expected to be less biased than the complete case analysis. Another limitation is that the participants were informed of their treatment allocation and the observed reduction in AD after publication of the results from the 2 year follow-up in 2010 [26], although we do not believe that this to have significantly affected the current results. Firstly, the knowledge of treatment allocation has not affected participant behaviour with equal numbers from the probiotic and placebo groups attending the clinical follow-up at 6 years. Secondly, the UKWP diagnosis was based on assessment by research nurses who were unaware of treatment allocation.

## Conclusions

In conclusion, we have previously shown that perinatal maternal probiotic supplementation is effective in reducing the cumulative incidence of AD in children up to 2 years of age. The current study does not conclusively demonstrate an ongoing benefit, however there is a strong trend towards a reduced cumulative incidence at 6 years. This would suggest that perinatal probiotics prevent and do not mere delay the onset of AD in childhood. The cumulative incidence of ARC and prevalence of asthma and atopic sensitisation were unaffected by the probiotic regime at 6 years of age.

## Additional file

Additional file 1: Includes (a) Table S1. Definition of asthma and allergic rhinoconjunctivitis used at 2 and 6 years; (b) **Table S2:** Baseline data, characteristics and allergy related diseases at 2 years for those who did and did not submit 6 year child health questionnaire; (c) Details of multiple imputation model and (d) Details of pattern mixture model sensitivity analysis.

## Abbreviations

AD: Atopic dermatitis
ARC: Allergic rhinoconjunctivitis
ProPACT: Probiotics in the Prevention of Allergies among Children in Trondheim
sIgE: Specific immunoglobulin E
SPT: Skin prick test
UKWP: UK Working Party diagnostic criteria for atopic dermatitis
ISAAC: International Study of Asthma and Allergies in Childhood
ITT: Intention to treat
MICE: Multiple imputation by chained equations
MAR: Missing at random
MNAR: Missing not at random
PMM: Pattern mixture model
